# Risk-adapted single or fractionated stereotactic high-precision radiotherapy in a pooled series of nonfunctioning pituitary adenomas: High local control and low toxicity

**DOI:** 10.1007/s00066-014-0715-0

**Published:** 2014-08-05

**Authors:** Jan Patrick Boström, Almuth Meyer, Bogdan Pintea, Rüdiger Gerlach, Gunnar Surber, Guido Lammering, Klaus Hamm

**Affiliations:** 1 Department of Neurosurgery, HELIOS Klinikum Erfurt, Nordhäuser Strasse 74, 99089 Erfurt, Germany; 2 Department of Endocrinology, HELIOS Klinikum Erfurt, Nordhäuser Strasse 74, 99089 Erfurt, Germany; 3 Department of Radiosurgery, HELIOS Klinikum Erfurt, Nordhäuser Strasse 74, 99089 Erfurt, Germany; 4 Department of Radiosurgery and Stereotactic Radiotherapy, MediClin Robert Janker Clinic and MediClin MVZ Bonn, Villenstrasse 8, 53129 Bonn, Germany; 5 Department of Radiotherapy and Radiation Oncology, Heinrich-Heine-University of Duesseldorf, Moorenstrasse 5, 40225 Duesseldorf, Germany; 6 Department of Neurosurgery, University Hospital of Bonn, Sigmund-Freud-Strasse 25, 53105 Bonn, Germany

**Keywords:** Nonsecretory pituitary adenoma, Radiosurgery, Stereotactic radiotherapy, Hypopituitarism, Hormoninaktive Hypophysenadenome, Radiochirurgie, Stereotaktische Radiotherapie, Hypophyseninsuffizienz

## Abstract

**Purpose:**

The purpose of this work was to evaluate a prospectively initiated two-center protocol of risk-adapted single-fraction (SRS) or fractionated radiotherapy (SRT) in patients with nonsecretory pituitary adenomas (NSA).

**Patients and methods:**

A total of 73 NSA patients (39 men/34 women) with a median age of 62 years were prospectively included in a treatment protocol of SRS [planning target volume (PTV) < 4 ccm, > 2 mm to optic pathways = low risk] or SRT (PTV ≥ 4 ccm, ≤ 2 mm to optic pathways = high risk) in two Novalis® centers. Mean tumor volume was 7.02 ccm (range 0.58–57.29 ccm). Based on the protocol guidelines, 5 patients were treated with SRS and 68 patients with SRT.

**Results:**

Median follow-up (FU) reached 5 years with 5-year overall survival (OS) of 90.4 % (CI 80.2–95 %) and 5-year local control and progression-free survival rates of 100 % (CI 93.3–100 %) and 90.4 % (CI 80.2–95 %), respectively. A post-SRS/SRT new visual disorder occurred in 2 patients (2.7 %), a new oculomotor nerve palsy in one pre-irradiated patient, in 3 patients (4.1 %) a pre-existing visual disorder improved. New complete hypopituitarism occurred in 4 patients (13.8 %) and in 3 patients (25 %) with pre-existing partial hypopituitarism. Pituitary function in 26 % of patients retained normal. Patients with tumor shrinkage (65.75 %) had a significantly longer FU (*p* = 0.0093). Multivariate analysis confirmed correlation of new hypopituitarism with duration of FU (*p* = 0.008) and correlation of new hypopituitarism and tumor volume (*p* = 0.023). No significant influence factors for occurrence of visual disorders were found.

**Conclusion:**

Our SRS/SRT protocol proved to be safe and successful in terms of tumor control and protection of the visual system, especially for large tumors located close to optic pathways.

## Abstract (de)

**Ziel:**

Evaluation eines prospektiv angelegten Behandlungsprotokolls einer risikoadaptierten Radiochirurgie (SRS) oder stereotaktischen Radiotherapie (SRT) von Patienten mit hormoninaktiven Hypophysenadenomen (NSA) aus zwei Zentren.

**Patienten und Methoden:**

73 NSA-Patienten (39 Männer, 34 Frauen) mit einem medianen Alter von 62 Jahren wurden nach einem prospektiven Protokoll entweder mit SRS (PTV < 4 ccm, > 2 mm zum optischen System = geringes Risiko) oder fraktionierter SRT (PTV ≥ 4 ccm, ≤ 2 mm zum optischen System = hohes Risiko) in zwei Novalis^®^-Zentren behandelt. Das mittlere Tumorvolumen betrug 7,02 ccm (Spanne 0,58–57,29 ccm). Nach diesem Protokoll wurden 5 Patienten mit SRS und 68 mit SRT behandelt.

**Ergebnisse:**

Ein medianes Follow-up von 5 Jahren wurde mit einer 5-Jahres-Überlebensrate von 90,4 % (CI 80,2–95 %) und einer lokalen 5-Jahres-Kontrollrate und progressionsfreier Überlebensrate von 100 % (CI 93,3–100 %) bzw. 90,4 % (CI 80,2–95 %) erreicht. Bei 2 Patienten (2,7 %) trat eine post-SRS/SRT-Störung des optischen Systems und bei einem auswärtig vorbestrahlten Patienten eine neue Okulomotoriusparese auf; bei 3 Patienten (4,1 %) wurde eine Sehverbesserung beobachtet. Eine neue komplette Hypophyseninsuffizienz post-SRS/SRT wurde in 4 (13,8 %) und in 3 Patienten (25 %) mit vorbestehender partieller Hypophyseninsuffizienz beobachtet. Bei 26 % der Patienten blieb die Hypophysenfunktion normal. Patienten mit Tumorschrumpfung (65,75 %) hatten ein deutlich längeres Follow-up (*p* = 0,0086). Die multivariate Analyse bestätigte eine Korrelation von neuer Hypophyseninsuffizienz mit der Follow-up-Dauer (*p* = 0,007) und eine Korrelation einer neuen Hypophyseninsuffizienz mit dem Tumorvolumen (*p* = 0,019). Signifikante Einflussfaktoren für das Auftreten einer Sehstörung wurden nicht identifiziert.

**Schlussfolgerung:**

Unser SRS/SRT-Behandlungsprotokoll erwies sich vor allem bei großen Tumoren in der Nähe des optischen Systems als erfolgreich und sicher hinsichtlich Tumorkontrolle und Schutz des optischen Systems.

## Abbreviations

PA: Pituitary adenoma
NSA: Nonsecretory pituitary adenoma
SRS: Stereotactic radiosurgery
SRT: Stereotactic radiotherapy
OS: Overall survival
PFS: Progression free survival
PTV: Planning target volume
CTV: Clinical target volume
RTOG: Radiation therapy oncology group
OARs: Organs at risk
LINAC: Linear accelerator
MRI: Magnetic resonance imaging
RT: Radiotherapy
Gy: Gray
CTC: Common Terminology Criteria for adverse events
RECIST: Response Evaluation Criteria in Solid Tumors
TSH: Thyroid-stimulating hormone
fT4: Free thyroxin
ACTH: Adrenocorticotropic hormone
LH: Luteinizing hormone
FSH: Follicle-stimulating hormone
IGF-1: Insulin-like growth factor 1
(hf) SRT: hypofractionated stereotactic radiotherapy
N.D.: not done

Pituitary adenomas (PA) are divided into secretory and nonsecretory varieties and the intention of treatment differ for the two entities. Treatment for secretory PA mainly aims to prevent excessive secretion of hormones, whereas treatment of nonsecretory PA (NSA) is intended to control tumor growth and prevent or reverse visual disorders and endocrinopathies [8, 12, 27]. So far, most articles have been summarizing both entities together; however we consider these two varieties in terms of clinic as well as in terms of treatment completely differently, which underlines the need to investigate these two varieties entirely separately. Thus, we excluded the hormone secreting tumors from the study presented herein.

Approximately 30 % of PA are nonsecretory and especially when causing visual symptoms are treated primarily with transsphenoidal surgery or craniotomy. Yet frequently patients have residual postoperative tumors and several studies have reported recurrences in about 20–50 % of cases treated with surgery alone [3, 6, 13, 28].

There are still no clear guidelines with regard to radiotherapy (RT) because of the lack of randomized controlled studies. RT is commonly considered in cases in which a large amount of tumor is left behind or if the residual tumor is located close to the optic nerves/chiasm and regrowth may lead to visual compromise. RT is also considered if residual or recurrent tumors invade in the cavernous sinus or in cases in which repeated surgeries have resulted in fibrosis and inoperability [1, 10, 31, 33, 36, 39]. A review on conventional radiotherapy for NSAs demonstrated an overall progression-free survival of 80–90 % at 10 years and 75–90 % at 20 years [25]. Considering the proximity of organs at risk (OARs) such as the optic nerves, chiasm and brain stem, the use of stereotactic irradiation has been increasingly considered [29, 36]. A review on stereotactic radiosurgery (SRS) for NSA reported a tumor growth control rate of 87–100 % with a follow-up of 6–60 months [26]. However, a single high-dose treatment may not be appropriate for large tumors or tumors adjacent to optic pathways because of the limited dose tolerance for these structures [37]. Thus, protection of optic pathways and the brain stem may be more efficiently achieved by using lower daily doses with a fractionated regime rather than SRS [17]. More recently, some reports have indicated promising outcomes with stereotactic radiotherapy (SRT) using conventional fractionation or moderate hypofractionation [5, 15, 16, 23]. However, these data are still preliminary with a relatively short follow-up.

In the study presented herein, however a high number of NSA has been prospectively followed after fractionated SRT and SRS with complete radiological, endocrinological, and ophthalmological data. All patients had been treated with harmonized protocols at two institutions with the same irradiation system either as SRS or SRT in a risk-adapted manner.

## Methods and patients

### Patients and treatment protocol

We included 73 patients with NSA from July 2000 to March 2010 fulfilling following eligibility criteria: (1) histologically confirmed or image diagnosed PA with endocrinological findings indicating NSA; (2) recurrent cases, patients receiving postoperative adjuvant SRS/SRT, inoperable patients, and patients who refused surgical resection; (3) no prior RT or chemotherapy for other cranial disease; and (4) willingness to provide written informed consent. The patient and tumor characteristics are summarized in Table 1.

**Table 1:** Overview of clinical data before and after irradiation and irradiation parameters

	Variable	Overall	Average
*N*		73	
*Age* (range)		(30–82)	60 (mean)
*No. of surgeries* (range)		(0–4)	1.41 (mean)
**Fractionation**			
	SRS	5	7 %
	SRT	67	92 %
	(hf) SRT	1	1 %
*RT*	Adjuvant	63	86 %
	primary	10	14 %
*CTV* (range), ccm		(0.58–57.29)	7.02 (mean)
	SRS	(1.04–1.94)	1.69 (median)
	SRT	(0.58–57.29)	4.05 (median)
	(hf) SRT	2.21	
*Fx* (range)		(1.00–31.00)	
	SRS	1	
	SRT	25–31	26 (mean)
	(hf) SRT	7	
**Daily dose**, Gy			
	SRS	15, 18, 20	
	SRT	1.8–2.0	
	(hf) SRT	5	
*Total dose* (range), Gy		(15.00–56.00)	
	SRS	18, 20, 30	
	SRT	45–62	52 (median)
	(hf) SRT	35	
*Follow up* (range) years		(0.5–11.0)	5.16 (mean)
**Tumor size** (at last follow-up)			
	Smaller	48	66 %
	Stable	25	34 %
	Larger	0	0 %
	N.D.	0	
**Hypopituitarism** (before RT)			
	Full	32	43.8 %
	Partial	12	16.4 %
	None	29	39.7 %
	N.D.	0	
**Hypopituitarism** (after RT)			
	Full	39	53.4 %
	Partial	15	20.5 %
	None	19	26.0 %
	N.D.	0	
**Dysfunction of optical system** (before RT)			
	Yes	36	49.3 %
	None	37	50.7 %
**Dysfunction of optical system** (after RT)			
	None	36	49.3 %
	Idem	31	42.5 %
	Improvement	3	4.1 %
	Aggravation	3	4.1 %

RT radiotherapy, SRS stereotactic radiosurgery, SRT stereotactic radiotherapy, (hf) SRT hypofractionated stereotactic radiotherapy, CTV clinical target volume, Gy Gray, N.D. not done

The treatment algorithm followed at the two institutions was as follows: SRS was considered the preferred treatment, if the target volume was smaller than 4 ccm and the closest distance to the optic pathways was above 2 mm. The single dose given was 15–20 Gy prescribed to the 80 % isodose line. In all other cases, SRT was preferred, consisting of 25–31 fractions in 1.8–2.0 Gy daily doses. Based on the protocol guidelines we used, only 5 patients were treated with SRS and the majority of 68 patients with SRT (see Table 1). Patient immobilization, treatment planning, geometrical accuracy of and clinical experiences with the Novalis® system used here have been reported by several investigators [9, 11, 14].

### Follow-up evaluation and statistical analysis

After SRS/SRT, the patients were followed at 6 and 12 months during the first year, and at intervals of 12 months thereafter. Regular follow-up studies included clinical examination, brain MRI, visual perception tests, and examinations of hormonal levels.

The following hormones were routinely tested: thyroid-stimulating hormone (TSH), free tyroxin (fT4), adrenocorticotropic hormone (ACTH), cortisol, luteinizing hormone (LH), follicle-stimulating hormone (FSH), testosterone, prolactin, and insulin-like growth factor 1 (IGF-1). A 24-h cortisol urine test was performed only in some cases. Any deviation from the age and gender adjusted hormone levels of the corticotrope, thyrotrope, and gonadotrope hormone axes was counted as hypopituitarism, a hypofunction of all hormone axes represents a complete insufficiency, an isolated somatotrope dysfunction before or after irradiation was not counted as a partial hypopituitarism. New deficits 3 months after radiotherapy were regarded as being associated with radiotherapy.

The local responses to RT were classified according to the Response Evaluation Criteria in Solid Tumors (RECIST). If there were no computer-generated tumor volumes available on follow-up MRI, tumor progression was defined as an increase in mean tumor dimension of more than 2 mm persisting on 2 or more consecutive studies. Tumor response was defined as decrease in mean tumor dimension of more than 2 mm persisting on 2 or more consecutive studies. Stable tumor was defined as no change in size or change of 2 mm or less. In most cases the DICOM data of the follow-up MRIs were available, so we could perform an image fusion with the planning MRI, generate and compare the tumor volumes, where response was defined as minus > 20 % volume and progression as plus 20 %.

The rates of overall survival, local control, and progression-free survival were calculated using the Kaplan–Meier method. Stepwise multiple linear regression tests were used in the analysis of following potential influence factors for tumor shrinkage, new hypopituitarism, or new visual deficit: age at treatment, SRS vs. SRT, primary vs. adjuvant SRS/SRT, CTV, total dose, number of fractions, follow-up in years, number of surgeries. Statistical analysis was performed using SPSS® (SPSS Inc., Chicago, IL, USA) software.

## Results

Of the 73 patients, 10 patients were irradiated without primary surgery (all of them received SRT), the remaining 63 patients received irradiation to a progressive and/or residual tumor after one or more surgeries. Most patients had one or two surgeries before irradiation, namely 36 and 17, respectively. There were 5 patients who had 3 and even 5 patients who had 4 prior surgeries.

SRS received 5 patients with small sized residual tumors (all CTVs < 2 ccm) with additional sufficient distance from the optical system (> 2 mm). Only one patient received a hypofractionated (hf) SRT with 7 fractions of 5 Gy, because the CTV was too large for SRS, but the distance from the optical system was large enough for the higher daily dose. SRT was prescribed in the 67 remaining patients (see Table 1).

**Fig. 1:**
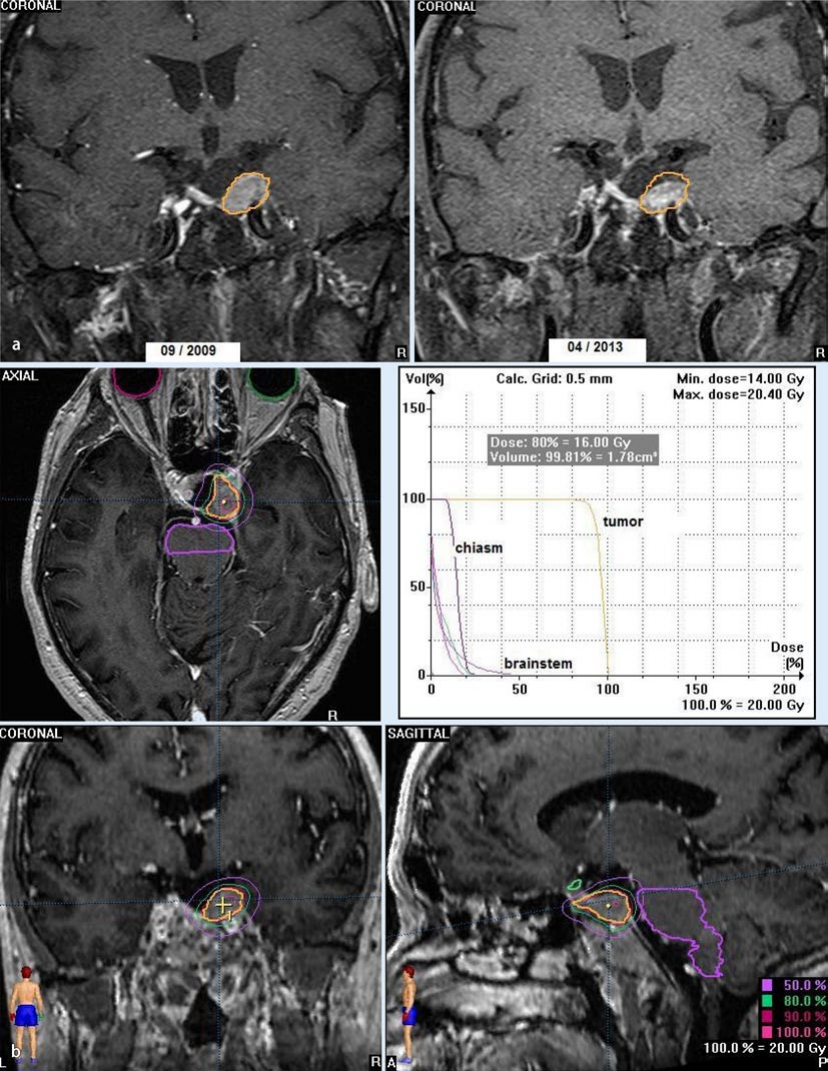
**a**, **b** MRI of patient (male, 72 years) with tumor progression after 2 transsphenoidal surgeries (the last in 2005), SRS with 20 Gy in March 2009 (CTV 1.88 ccm). At 4-year follow-up, tumor regression, no visual disorder, no hypopituitarism

**Fig. 2:**
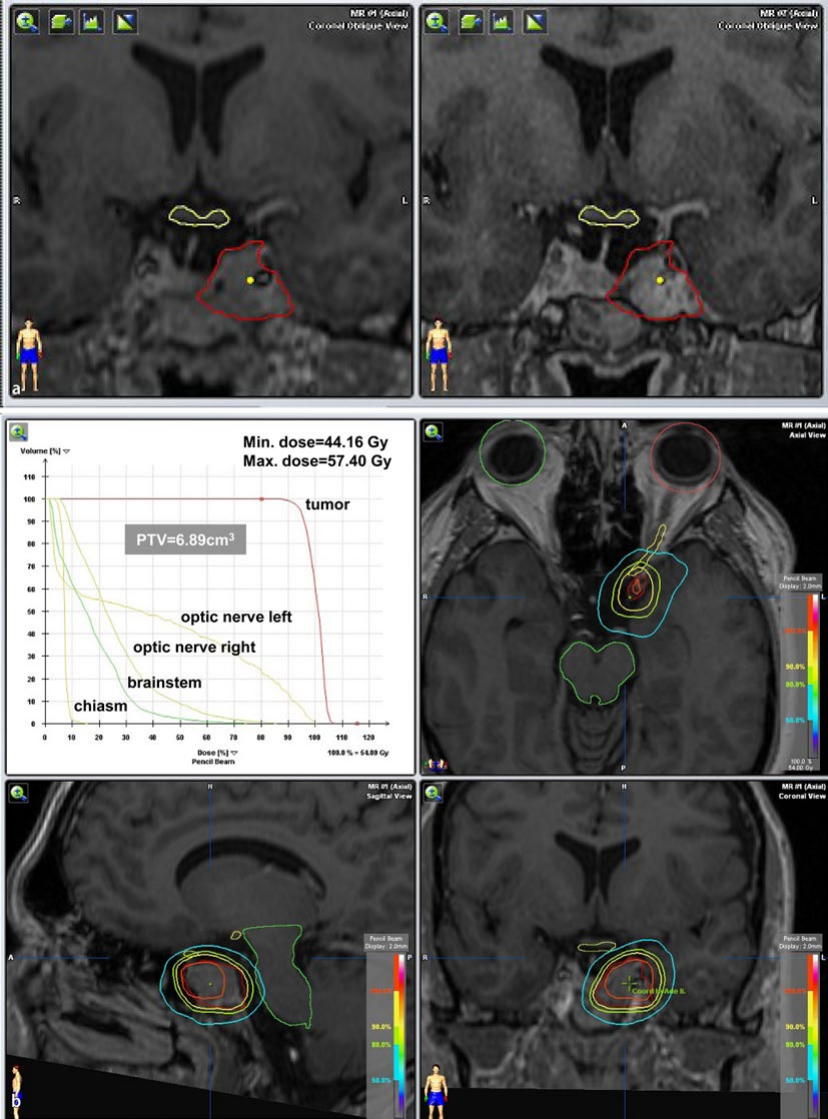
**a**, **b** MRI of patient (male, 41 years) with tumor progression after 2 transsphenoidal surgeries (the last in 2007), SRT with 5 × 1.8 ad 54 Gy in 2009 (CTV 5.13 ccm). At 3-year follow-up, tumor regression, no visual disorder, hypopituitarism (partial) idem

The figures show examples of treatment planning’s with dose–volume histograms, isodose lines and OARs including the chiasm, optic nerves, and brain stem as well as MRI follow-ups in a typical SRS (Fig. 1a **and** b) and a typical SRT case (Fig. 2a **and** b).

### Survival and local control

Follow-up varied from 0.5–11 years (median, 5 years). A total of 69 patients were followed at least 1 year. There were 48 patients (65.75 %) who showed a response with tumor shrinkage, 25 patients (34.25 %) with stable disease, and no patient showed progressive disease at the latest follow-up (Table 1).

The 5-year overall survival rate reached 90.4 % (95 % confidence interval [CI] 80.2–95 %). We have seen no local recurrences in our series. The 5-year local control and progression-free survival rates were 100 % (CI 93.3–100 %) and 90.4 % (CI 80.2–95 %), respectively. The difference between the values is due to disease-unrelated death of some patients.

### Side effects and complications

A post-SRS/SRT visual disorder was observed in 2 patients (2.7 %; one worsening of a visual field disturbance and one vision acuity decrease in a previously elsewhere irradiated patient). However, there was also an improvement of a visual disorder in 3 patients (4.1 %; visual field disturbance in all and one additional vision acuity improvement in one patient with a primary SRT). For overview see Table 2.

**Table 2:** Overview of the patients with improvement or aggravation of dysfunction of the optical system after RT

Patients	CTV (ccm)	RT	Surgeries (n)	Follow-up (years)	Dysfunction before RT	Outcome
RH, m, 69 years	2.46	SRT	1	6	None	New oculomotor nerve palsy; serveral other causes possible: dilatative angiopathy, ischaemic neuropathy, neurofibromatosis type 2
TH, m, 61 years	2.49	SRT	2	9	Visual field	(mild) Aggravation
BM, f, 72 years	7.73	RT and SRT	2	1	None	New visual acuity impairment left (improvement after cortisone); preexisting amaurosis right after surgery; conventional pre-irradiation in 1996 and 04–05/2005 SRT; cardiac death 18 months after SRT
GR, f, 72 years	3.47	SRT	1	11	Visual field	Improvement
TU, f, 62 years	19.81	(primary) SRT	0	4	Visual acuity/visual field	Improvement (visual field >visual acuity)
ER, m, 64 years	7.66	SRT	2	7	Visual acuity/visual field	Improvement (visual field)

f female, m male, RT radiotherapy, SRT stereotactic radiotherapy, CTV clinical target volume

Only 29 of 73 patients (39.7 %) had normal pituitary function before SRS/SRT. Twelve of 73 patients (16.4 %) had partial dysfunction before SRS/SRT.

Post-SRS/SRT new complete hypopituitarism was observed in 4 patients who received no hormone replacement therapy after surgery (13.79 %) and in 3 patients with partial hypopituitarism after surgery (25 %). Post-SRS/SRT new partial hypopituitarism was observed in 4 patients (13.79 %). In one patient partial hypopitutarism had normalized after SRT. A total of 19 of 29 (66 %) of the patients with normal function before SRS/SRT remain with a normal pituitary function after SRS/SRT. No radiation-induced brain necrosis and only one new paralysis of an oculomotor nerve in a patient with additional neurofibromatosis was observed, whereby the causal relationship of the oculomotor nerve palsy with the irradiation could not be proven (see Table 2).

### Univariate and multivariate analysis

Patients with tumor shrinkage (*n* = 48) had compared to patients without tumor shrinkage (*n* = 25) a significantly longer follow-up (6 vs. 4 years, t-test, *p* = 0.0093). The positive correlation between tumor shrinkage and duration of follow-up remains significant in the stepwise (backward and forward) multivariate analysis of the data (*p* = 0.020).

Patients with new endocrine deficits had a significantly longer post-radiation follow-up-time compared to patients without new endocrine deficits (7 vs. 4 years, t-test, *p* = 0.0001). The stepwise multivariate analysis (backward and forward) of the data confirmed a correlation of new endocrine deficits with the duration of follow-up (*p* = 0.008 and *p* = 0.006, respectively) meaning that the longer the follow-up period goes, the more likely a pituitary hypofunction appears. Additionally a weak positive correlation of new hypopituitarism with the CTV was found (only stepwise backward, *p* = 0.023).

No correlation between a new post-SRS/SRT visual damage and any of the predictors in the multivariate analysis was detected.

## Discussion

In this two center study, we prospectively treated a relatively high number of NSA with a comparably long follow-up and complete radiological, endocrinological and ophthalmological data at the last follow-up. In addition all patients were irradiated with the same irradiation system either as SRS or SRT in a harmonized risk-adapted protocol achieving a high 5-year local control (100 %) and progression-free survival rate (90.4 %) with low toxicity. A post-SRS/SRT visual disorder occurred only in 2 patients (2.7 %), the only other neurological deficit was a new oculomotor nerve palsy which occurred in a pre-irradiated patient. The rate of 39.7 % patients with normal pituitary function before radiotherapy decreased to 26 % after radiotherapy.

While concerning conventional irradiation [3, 7, 21, 25, 32, 33] and also classical radiosurgery with gamma knife or LINAC [2, 22, 31, 34–36], case series with long-term follow-up were already published, these have been lacking so far for fractionated stereotactic radiotherapy. This is understandable, since this technique has only been used regularly since the end of the 1990s [4, 16]. In the last 5 years, the first of such studies have been published [18–20, 24, 30, 38]. First, we want to put our data in respect of three in our view relevant series published in recent years.

In 2007 Kong et al. [18] published a retrospective cohort study of 125 patients including 54 hormone secreting PA. Of the 71 NSA, 42 were treated with LINAC SRT (48–54 Gy) and 29 tumors were treated with Gamma knife SRS (20–28 Gy). The mean follow-up was 36.7 months. They presented indeed a large case number, but also included patients with secretory adenomas. We have excluded all those cases to avoid the bias of different therapeutic approaches (higher radiation dose and antihormonal adjuvant therapy in secretory adenomas) in these two types of pituitary adenomas. Nevertheless, our case number of 73 patients with NSA and a median follow-up of 5 years compares favorably. Kong et al. found an overall actuarial progression-free survival rate of 97 % at 4 years. We can confirm the long-term high tumor control and progression-free rate of more than 90 %. A differentiated comparison with our data regarding the long-term effects on damage of the optical system or the pituitary does not appear reasonable since in the study of Kong et al. no objective visual testing was done in the majority of patients and no differentiation between complete and partial insufficiency was undertaken.

In 2013 Kopp et al. [20] reported their experience with 37 PA including 29 NSA. In their series all patients had surgery before SRT; the median follow-up was 57 months. The tumor control rate was 91.9 % with tumor size unchanged in 59.5 % and only 3 (8.1 %) progressions in two hormone secreting tumors. Visual acuity improved in 7 cases (19 %) and deteriorated in 2 cases (5 %). Visual field improved in 1 and worsened in 1 patient (both 2.7 %). The pituitary function of 22 % was normal after SRT (24 % before SRT), all other patients had a partial (43 %) or complete dysfunction (35 %). This is largely in line with our results. A total of 19 of 73 patients (26 %) remain with a normal pituitary function after (mostly one or more surgeries plus) SRS/SRT in our series. Whereas post-SRS/SRT new complete hypopituitarism was observed in 4 patients who received no hormone replacement therapy after surgery (13.79 %), the rate of complete hypopituitarism in patients with partial hypopituitarism before SRS/SRT was even higher (25 %). We can confirm a very low rate of new damage of the optical system. In our series there were only 2 patients (2.7 %; one worsening of a visual field disturbance and one vision acuity decrease in a previously elsewhere irradiated patient). But we also observed an improvement of a visual disorder in 3 patients (4.1 %). These results are also in line with the data of Kocher et al. [17] reporting about SRT of perioptic tumors. Regarding risk factors for occurrence of a damage of the optical system, we found both in the univariate and in the multivariate analysis, no statistically significant influence factors.

Rieken et al. [30] reported in 2013 about their experience with 92 patients with pituitary adenomas including 55 NSA. RT was conducted using either 3D conformal or fractionated stereotactic techniques (76 patients). Median follow-up was 152.5 months. Before treatment, 2 % of all patients were diagnosed with adenoma-related hypopituitarism. Following surgery, 68 % suffered from new pituitary deficits. RT was associated with 5.4 % new visual deficits and 10.9 % new hypopituitarism in their series. PFS following RT was 90.4 and 75.5 % at 120 and 240 months. Despite their long follow-up the new hypopituitarism rates are low, but again in the long term only 20–25 % of the patients retain a normal pituitary function. In our series we could demonstrate a significant correlation between length of follow-up and occurrence of new hypopituitarism.

Possible differences of the incidence of hypopituitarism after (S)RT or SRS [40] may reflect different patient selection and length of follow-ups, also our data provide no conclusive information about this aspect and large comparative prospective studies are needed to clarify this issue. If there is a trend, then it is a correlation with tumor volume and length of follow-up. That adenomas with a large volume are supplied mostly to fractionated (stereotactic) irradiation would then have to be taken into account.

In our series no secondary cancer was observed. Since our follow-up times are fairly long, we are able to confirm that secondary malignancies are no primary concern in the treatment of pituitary adenoma patients.

In the absence of comparative studies, the choice of the radiation technique should be based on tumor characteristics. SRS is usually offered to patients with relatively small adenomas with a distance of at least 2 mm from the optic system. SRT should be preferred in patients with large tumors in close proximity of the optic apparatus, since the treatment is delivered within the radiation tolerance limits of cranial nerves, including the optic apparatus.

Our experience demonstrated that the Novalis® system is very versatile in delivering SRS or SRT so that both modalities can be offered and one can search for the best individual solution.

## Limitations

Because this study is a two center study, the data presented are not representative, and the results cannot be generalized. However, the study can still be of value as a sample study submitted by two major departments of radiosurgery and stereotactic radiotherapy.

## Conclusion

After 10 years of experience, we consider our risk-adapted protocol of radiosurgery or fractionated SRT depending on tumor volume and distance between tumor and the optical system as safe and successful in terms of tumor control and protection of the visual system, especially for large tumors and tumors located near to the optic pathways. However, one has to accept an increased rate of new hypopituitarism.

## Compliance with ethical guidelines

### Conflict of interest

J.P. Boström, A. Meyer, B. Pintea, R. Gerlach, G. Surber, G. Lammering, and K. Hamm state that there are no conflicts of interest.

All studies on humans in the present manuscript were carried out with the approval of the responsible ethics committee with national law and the Helsinki Declaration of 1975 (in its current, revised form). Informed consent was obtained from all patients included in studies.
